# Transitions in care during the end of life: changes experienced following enrolment in a comprehensive palliative care program

**DOI:** 10.1186/1472-684X-4-3

**Published:** 2005-02-22

**Authors:** Frederick I Burge, Beverley Lawson, Patrick Critchley, David Maxwell

**Affiliations:** 1 Department of Family Medicine, Dalhousie University, Halifax, NS, Canada; 2 Active staff, Timmims and District Hospital, Timmons, ON, Canada

## Abstract

**Background:**

Transitions in the location of care and in who provides such care can be extremely stressful for individuals facing death and for those close to them. The objective of this study was to describe the distribution of transitions in care experienced by palliative care patients following admission to a comprehensive palliative care program (PCP). A better understanding of these transitions may aid in reducing unnecessary change, help predict care needs, enhance transitions that improve quality of life, guide health care system communication links and maximize the cost-effective utilization of different care settings and providers.

**Methods:**

Transition and demographic information pertaining to all patients registered in the PCP at the Queen Elizabeth II Health Sciences Centre (QEII), Halifax, Nova Scotia, Canada between January 1, 1998 and December 31, 2002 and who died on or prior to December 31, 2002 was extracted from the PCP database and examined. A transition was defined as either: (1) a change in location of where the patient was cared for by the PCP or, (2) a change in which clinical service provided care. Descriptive analysis provided frequencies and locations of transitions experienced from time of PCP admission to death and during the final two and four weeks of life, an examination of patient movement and a summary of the length of stay spent by patients at each care location.

**Results:**

Over the five year period, 3974 adults admitted to the QEII PCP experienced a total of 5903 transitions (Mean 1.5; standard deviation 1.8; median 1). Patients with no transitions (28%) differed significantly from those who had experienced at least one transition with respect to survival time, age, location of death and diagnosis (p < 0.0001). The majority of patients were admitted to the PCP from various acute care units (66%). Although 54% of all transitions were made to the home, only 60% of these moves included care provided by PCP staff. During the last four weeks of life, 47% of patients experienced at least one transition; 36% during the final two weeks of life. Shorter stays in each location were evident when care was actively provided by the PCP.

**Conclusion:**

A relatively small number of patients under the care of the PCP at the end of life, made several transitions in care setting or service provider. These particular patients need closer scrutiny to understand why such transitions take place so that clinical programs may be designed or modified to minimize the transitions themselves or the impact transitions have on patients and families.

## Background

For individuals facing death, and for those close to them, transitions in the location of care and in who provides care can be extremely stressful.[1] Such transitions include moving from home to hospital or to long-term care facilities, from ward to ward within hospitals, or in and out of care directed by particular care providers (such as specialists). With or without good continuity of information transfer, patients and caregivers may, at each transition, need to retell their story, renegotiate the goals of care and redefine their relationships with health professionals. At each point, new communication channels must be established and new trusts formed.

Much of the health service research literature in end-of-life care focuses on issues either in hospital or at home. [2-5] We believe it is also important to examine the changes or transitions patients make in where they are cared for and by whom during the end-of-life.

The ultimate goal of understanding these transition issues better is to reduce unnecessary changes, help to predict care needs, enhance transitions that improve patient and caregiver quality of life, guide communication links within the health care system and maximize the cost-effective utilization of different care settings and providers.

The primary purpose of this study was to describe the distribution of transitions in care experienced by palliative care patients during the time subsequent to admission to a comprehensive palliative care program. We also report the proportion of transitions as death becomes imminent (the last two and four weeks of life) and describe the length of stay in each location of care or care setting.

## Methods

### Subjects

Subjects included all adult patients registered in the palliative care program (PCP) at the Queen Elizabeth II Health Services Centre (QEII) in Halifax, Nova Scotia, Canada between January 1, 1998 and December 31, 2002 with a recorded date of death on or prior to December 31, 2002. The PCP includes multidisciplinary care for the dying, an in-patient acute care unit, an in-hospital consultation service, a home consultation service and an oncology outpatient clinic consultation service. The team consists of physicians, nurses, social workers, pharmacists, spiritual care and volunteers. There is no free standing hospice facility in Nova Scotia. The PCP has existed since 1988 and, by 1997, had at least one contact with over 62% of those who die annually of cancer in the Halifax Regional Municipality (population approximately 350,000).[6]

### Data

Individual level information extracted from the PCP database included demographics (sex, date of birth, date of death, postal code), diagnoses, the relationship of the primary caregiver to the patient (for example, spouse, daughter, son, friend), reason for referral, location of death and program transition data. The program transition information provided the date of each transition and locations the patient had been moved to or from (for example, home, an acute care facility, long-term care) as well as the clinical service providing care. For example, for inpatients the service might be the Palliative Care Unit, or a medical or surgical service; for outpatients it might be the Nova Scotia Cancer Centre (NSCC) PCP clinic, the PCP Home Support Service, or the family doctor. The service indicator field also provided a record of whether patients were 'actively' being cared for by PCP staff or whether their care had been transferred to the staff of the NSCC or family doctor (either locally or elsewhere in the province).

Ethical approval for this research was provided by the Nova Scotia Capital District Health Authority research ethics board.

### Measures

A transition in this study is defined as either: (1) a change in location of where the patient was cared for by the PCP or, (2) a change in which clinical service provided care. For example, a transition might be a move to or from the home, a specific acute care unit or a long-term care facility. A transition would also occur if the patient stayed in a single location, for example at home, but the care being provided was transferred from PCP staff ('actively cared for' by the PCP) to their family physician or home care nurse ('non-active' and no longer 'actively' cared for by PCP staff) or vice versa. This 'transfer of care' transition scenario is illustrated below:



Length of stay was calculated as the number of days a patient stayed in a single location while receiving the same form of care. For example, a patient might be admitted to the PCP from home and stay in the home for a total of 86 days at which time the patient was admitted to an acute care inpatient unit. After spending 10 days in acute care the patient was sent home where they died 25 days later.



In this scenario the patient experienced two transitions (1. from home to acute care; 2. from acute care to home) but had three stay periods (two at home and one in acute care).

Survival in this study was defined as the number of days between date of the initial admission to the PCP and death. Cancer is the most prevalent disease among PCP patients, in particular lung cancer. To reflect this we created a diagnostic summary with three categories: lung cancer, all other cancers and other disease only (no cancer). Lung cancer was separated from other cancers as it is the most common cancer affecting both sexes in Nova Scotia, has a short prognosis and often has PCP involvement.

### Analysis

The analysis focused on providing a description of the number and location of transitions experienced by patients over the five-year study period. For each patient, the total number of transitions occurring from the date of initial admission to the PCP to the date of death and during the final two and four weeks of life were counted and described. Locations of admission, death, care and service provision are summarized as well as an examination of patient movement from location to location or 'site movements'. Summary statistics are provided to describe the length of stay (LOS) or number of days spent by all patients at each care location.

Differences between patients who experienced no transitions versus those who experienced at least one transition were assessed using contingency tables with chi-square techniques and logistic regression.

## Results

In total, 3972 adult patients were admitted to the QEII PCP between January 1, 1998 and December 31, 2002 and had died on or prior to December 31, 2002. There was a slight preponderance of male patients (52%); patients tended to be older (mean 68.5 years, standard deviation [SD] 13.7) and diagnosed with cancer (90%) (Table 1). Lung was the major cancer site accounting for 29% of all cancer diagnoses. Survival time, the number of days between program admission and death, was highly variable, ranging from 0 days (died same day as admitted to the PCP) to 1688 days, with an average of 100.6 (SD 163.2) and median of 45 days. As recorded in Table 1, 40% of patients survived less than 15 days and 24% survived 121 days or more. Eighty five percent of patients admitted to the PCP survived 6 months or less.

**Table 1:** Characteristics of patients admitted to the Queen Elizabeth II Palliative Care Program between Jan 1, 1998 and Dec 31, 2002 and died during the same period

**Characteristic**	Number of patients* (%)
**Sex**	
Female	1925 (48.5)
Male	2047 (51.5)
**Age, years**	
< 60	992 (25.1)
60–69	873 (22.1)
70–79	1208 (30.6)
≥ 80	878 (22.2)
**Year of admission to PCP**	
1998	883 (22.2)
1999	824 (20.8)
2000	876 (22.1)
2001	771 (19.4)
2002	618 (15.6)
**Year of death**	
1998	645 (16.2)
1999	820 (20.7)
2000	858 (21.6)
2001	824 (20.8)
2002	824 (20.8)
**Survival (days)**	
0–30	1605 (40.4)
31–60	723 (18.2)
61–90	432 (10.9)
91–120	251 (6.3)
121+	960 (24.2)
**Location of death**	
Hospital death (not in PCP unit)	2009 (50.6)
Inpatient PCP unit	635 (16.0)
Home	1218 (30.7)
Long-term care facility	110 (2.8)
**Diagnoses, summarized**	
Lung cancer	1025 (26.1)
All other cancers	2525 (64.3)
Other disease, no cancer	375 (9.6)
**Caregiver relationship**	
Spouse	2165 (57.8)
Child	1017 (27.2)
Parents / other relations	448 (12.0)
Other	116 (3.1)
**Primary reasons for referral to PCP**	
(responses are not exclusive)	
Pain	1630 (41.0)
Other symptoms	1868 (47.0) 1729 (43.5)
Patient support	1729 (43.5)
Family support	1620 (40.8)
Staff support	317 (8.0)
Home consultation	1067 (26.9)
Terminal care	345 (8.7)
Respite care	5 (0.1)
Grief	19 (0.5)

* N = 3,972. Smaller n by characteristic due to missing values.

**Figure 1:**
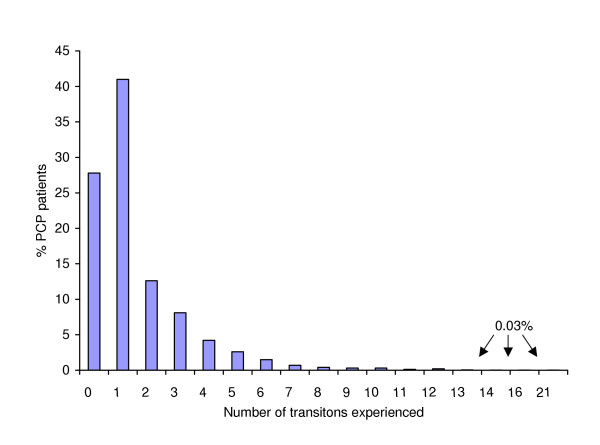
Total transitions over a five year period among all patients admitted to a comprehensive Palliative Care Program (PCP) (N = 3972)

Figure 1 illustrates the distribution of the number of transitions experienced by patients over the five-year study period. The number of transitions totalled 5903, ranging from 0 to 21, with an average of 1.5 (SD 1.8) per patient (median: 1). Overall, 28% did not experience a transition, 41% experienced one transition and 31% experienced two or more. Patients with no transitions experienced no change in the care provided or the location of care from the point of PCP admission to death. However, this group differed significantly from patients who experienced at least one transition, at the 0.001 level of significance, with respect to survival time, age, location of death and diagnosis. Compared to those who had at least one transition during the time period between admission to the PCP and death, patients with no transitions were much more likely to have a survival time of fourteen days or less (64% versus 10%), to be aged 80 years or older (32% versus 19%), to experience a hospital death (68% versus 44%) and to have a diagnosis other than cancer (19% versus 6%).

The majority of patients (66%) were initially admitted to the PCP as inpatients of various acute care units (n = 2619). Of this inpatient group, only 65 patients (2.5%) were admitted directly into the inpatient unit of the PCP. PCP admissions to care at home accounted for 34% of patients (n = 1335). Care services provided to home patients were provided upon admission by either PCP home care staff (51%) or on an outpatient basis through the NSCC (49%). Very few were admitted to the service from a long-term care facility (n = 17).

Overall, 54% of all transitions were made to the home, 27% to other acute care inpatient units within the same hospital, 17.5% to the PCP inpatient unit, and almost 2% to a long-term care facility. PCP staff members, however, were not always actively providing the care provided within each of these locations. For instance, in transitions to home only 60% involved care being 'actively' provided by PCP home care staff or through the NSCC PCP outpatient clinic. The reasons why the PCP was not involved in care provision are varied and include improvements in health status (for example their original symptoms have abated, or they were thought to be dying, but improved unexpectedly), changes in their personal caregiver situation or movement out of the PCP coverage area etc. In contrast, few transitions involving acute care units were due to a change in care from active care provision by PCP staff to non-active PCP care (4%). Table 2 illustrates the number of transition changes from one location or care site to another. For example, from the table we show that 32% of transitions originating from the home were to the PCP inpatient unit; 65% of transitions from the PCP inpatient unit were to the home.

**Table 2:** Location or site / care movements: a summary of the number of transitions made by all patients by location change

**Location / care changing FROM**	**Location / care changing TO Number of all transitions (%)**					
	PCP inpatient unit	All other acute care units	Home	Home with NSCC care	Long term care	Total
	
PCP inpatient unit	120^1 ^(26.1)	28 (6.1)	301 (65.4)	0	11 (2.4)	460 (7.8)
All other acute care units	184 (7.2)	350^2 ^(13.7)	1899 (74.5)	73 (2.9)	44 (1.8)	2550 (43.2)
Home	628 (32.1)	897 (45.8)	199^3 ^(10.2)	206 (10.5)	29 (1.5)	1959 (33.2)
Home (with NSCC care)	93 (10.4)	290 (32.3)	504 (56.1)	9^3 ^(1.0)	2 (0.22)	898 (15.2)
Long term care	3 (9.1)	10 (30.3)	14 (42.4)	0	6^3 ^(18.2)	33 (0.6)
Total	1028 (17.4)	1575 (26.7)	2914 (49.4)	288 (4.9)	92 (1.6)	5900^4 ^(100)

Site/care movements from and to the same location were evident.

^1 ^The patient was admitted under the PCP but placed in a bed elsewhere in the hospital while waiting transfer to the inpatient PCP unit. The movement in this situation reflects a physical location change from within the acute care facility to the PCP inpatient unit. It does not represent a change in care.

^2^Site movements across different acute care units are captured here.

^3^Each of these same site movements reflect a change in 'active' care status to 'non-active' care status or vice versa. 'Active' care is defined as care provided to a patient by PCP staff. 'Non-active' care refers to care being provided by individuals not associated with the PCP program.

^4^Three transition records were missing site information

During the last four weeks of life, 47% of patients experienced at least one transition. The majority of transitions were to an acute care facility (26% to the PCP inpatient unit, 35% to other acute care units), followed by the home (37%) and long-term care (2%). Similar patterns were evident when moves associated with the 2 weeks prior to death were examined. At least one transition was experienced by 36% of patients during these final 2 weeks of life. Moves to an acute care facility accounted for 68% (29% to the PCP inpatient unit; 39% to other acute care units), to the home 30% and 2% to long-term care.

The location of death among PCP patients followed a pattern similar to that found at admission. Sixty-seven percent (n = 2644) of deaths occurred within an inpatient acute care unit, 16% of these deaths occurred in the PCP inpatient unit (n = 423). Home deaths were experienced by 31% of all admissions (n = 1218), while 3% (n = 110) of patients died as a long-term care resident.

Table 3 summarizes the median length of stay (LOS) in days associated with each transition by location or care setting and the PCP's role in providing care. Medians are reported due to the very wide, skewed distributions associated with each LOS. We have split the LOS by PCP role in care to illustrate how LOS tended to be longer during transitions where PCP health care providers did not provide care. The median length of stay spent by patients who transitioned to acute care units without PCP care was 27.5 days. In contrast, transitions to the PCP inpatient unit and other acute care units providing active care by PCP health care providers was much shorter, with median days stayed of seven and six days respectively. The median length of stay spent at home while under the care of PCP home care providers was 28 days, 10 days less than that experienced at home when care was provided by others not associated with the PCP.

**Table 3:** Median length of stay spent in each care setting by PCP role in care^1^

**Location / care setting**	**Median number of days by PCP role in care (range)**	
	
	**'Active' care**	**'Non-active' care**
Acute care:		
PCP inpatient unit	7 (0 – 175)	-
All other inpatient units	6 (0 – 390)	27.5 (0 – 1139)
		
Home^2^	28 (0 – 845)	38 (0 – 1658)
Home, followed by NSCC	30 (0 – 1295)	44 (2 – 901)
		
Long-term care	18 (0–112)	63 (3 – 1079)

^1 ^'Active' care is defined as care provided to a patient by PCP staff.

'Non-active' care refers to care being provided by individuals others not associated with the PCP program.

^2 ^Home is defined as when a patient is not required to leave their home for contact with health care providers.

Home, followed by NSCC indicate the patient leaves the home to go to the PCP clinic at the Cancer Centre

## Discussion

Patients followed by the QEII PCP are primarily elderly (75% 60 years of age or older), urban dwellers, with cancer and survive less than six months from time of initial program admission (80%). This age and sex distribution is similar to that experienced among all Nova Scotians who died due to cancer between 1992 and 1998.[2] The average number of transitions experienced in care settings by this group of elderly was 1.5. Two or more transitions were experienced by 31% of patients. We were surprised that the vast majority of patients (69%) had fewer than two transitions. We had expected the number to be somewhat higher. Our clinical experience of providing care for these patients is perhaps skewed by the challenges faced by the minority of patients with multiple transitions.

Patients who had no transitions beyond entry to the PCP appear to be a different population than those with one or more transitions. This group may be a much sicker population since they tended to have much shorter survival times, were older and more likely to die in hospital. They were also more likely to die of disease other than cancer. This population warrants special attention in program planning and service delivery given their potentially higher institutional based needs.

The most common transition identified was from an acute care hospital unit "to" the home. This fact, in and of itself, provides evidence for the substantial focus that must take place in hospital on discharge planning for those at the end of life. Attention to the multiple discharge issues for this unique group of patients is likely the single biggest transition issue facing the acute care units and the consulting QEII PCP. Issues of symptom control, drug supplies, home equipment needs, family and professional caregiving needs in the home, advance planning for routine follow up and crisis management as well as the psychological supportive issues all need planning. The acute care teams must do this planning along with the QEII PCP consult team in collaborative fashion. These transitions present challenges in coordination and information transfer in order to facilitate continuity of care for patients and families.

The next most common category of transitions was from home "to" the hospital, either to an acute care unit or to the PCP inpatient unit. Just as the previous transition needs to be planned and coordinated so does the home to hospital one. Unfortunately we have no data on how this latter transition occurs. Most often it may be due to a symptom crisis in the home, a lack of caregiver capacity in the home, or a lack of financial resources to bring adequate care to the home.[7] Given the substantial pressure on acute hospital beds in Canada today, many of these admissions take place via the emergency department. Such environments may be appropriate in acute symptom stabilization before admission but the emergency department could be bypassed with planned and coordinated direct admission to the inpatient unit concerned. More information is needed on the "route" taken to hospital and the issues that require admission, the goals of admission and whether or not hospital based care versus respite / long-term care would best meet patient care needs.

Most patients came to the care of the PCP from within the acute care system. This may be a reflection of the lateness of referral for a substantial number of individuals (40% dying within 15 days of referral). These individuals, by the time of referral, may be quite ill in hospital. This initial entry to PCP (also a transition of care but one we did not explore) may need to be a focus of concern to better understand it. We need to understand the timing of referrals, what can be done to identify and meet patients' needs when required and whether these needs could be met outside of the hospital setting.

Once under the care of PCP the most common transitions were "to" home. Only a very small number of patient move "to" or "from" long term care (LTC). The fact that the majority of transitions were to home reflects the goals and expertise of the PCP in emphasizing the home location by coordinating the services needed for care at home. The fact that long term care is a rare transition also needs exploration. Policies may exist or wait times for admission may be such that transition to these facilities for people with short prognosis is difficult to achieve. At the same time, few patients transition "from" long term care. Some local LTC facilities have intramural palliative care programs designed to specifically meet the needs of their residents and their goals of staying in LTC as death approaches, avoiding hospital transfers.

The results of this study indicate almost half of patients have a transition in the last month of life and 36% in the last 2 weeks of life. As death approaches these transitions are more likely to be "to" hospital (i.e. 62% of those in the last 4 weeks and 68% of those in the last 2 weeks). Defining the "appropriateness" of these late transitions is difficult. As stated before, they may be due to acute symptom crises or caregiver inability to meet all of the care needs. We have also heard that patients are admitted when they appear in emergency departments with these issues as they have not been able to access professionals to assess and problem solve during nights and weekends.[7] Therefore, some late admissions are entirely appropriate and others may have been avoidable if more resources were available to patients and families at home.

As to the generally longer stays for patients when not under "active PCP" care, it may be that they are much less sick with other chronic, non-palliative problems and their prognosis is longer. One might also postulate that for the in-patients, the active involvement of PCP may facilitate shorter stays and transitions to the home. Such facilitation could include more rapid control of symptoms or more expeditions discharge planning. The phenomena may be similar in both the home and long-term care settings.

### Limitations

One substantial limitation is the loss of information pertaining to patients who are transitioned to non-active care permanently. Although we do have a record of their death, once a patient is moved to non-active care and ceases further involvement with the PCP, we do not have a record of who has taken responsibility for their care or where this care has been received. Our results and our clinical experience suggest this group may be quite different from those who remain actively cared for by the PCP. We have begun efforts to collect information about this group in order to understand them better.

## Conclusion

In conclusion, a small number of patients under the care of the PCP very near the end of their lives, make several transitions in care setting or service provider. These particular patients need much closer scrutiny in order to understand why such transitions take place. We will then be able to design or modify clinical programs to minimize the transitions themselves or the impact the transitions have on patients and families. Possible negative impacts of multiple transitions include discontinuity of care, poor coordination of care, financial burden and psychological stress that each move may bring to patients and their families.

## Competing interests

The author(s) declare that they have no competing interests.

## Authors' contributions

FB and BL participated in the conceptualization and design of the project, the analysis and interpretation of the data, first-drafted the majority of the article, and incorporated co-authors' comments into the final draft. PC participated in the conceptualization and design of the project, interpretation of data, and revising of the manuscript. DM participated in the analysis and interpretation of data and revising each draft for critical content. All authors gave approval to the final version.

## Pre-publication history

The pre-publication history for this paper can be accessed here:


